# Reinforcement of pimobendan with guideline-directed medical therapy may reduce the rehospitalization rates in patients with heart failure: retrospective cohort study

**DOI:** 10.1186/s40780-024-00346-w

**Published:** 2024-05-20

**Authors:** Yoshiki Iwade, Yoshiaki Kubota, Daisuke Hayashi, Takuya Nishino, Yukihiro Watanabe, Katsuhito Kato, Shuhei Tara, Yuya Ise, Kuniya Asai

**Affiliations:** 1 Department of Pharmaceutical Service, Nippon Medical School Hospital, 1-1-5 Sendagi, Bunkyo-ku, Tokyo, 113-8603 Japan; 2 Department of Cardiovascular Medicine, Nippon Medical School, 1-1-5 Sendagi, Bunkyo-ku, Tokyo, 113-8603 Japan; 3 Department of Health Care Administration, Nippon Medical School, 1-1-5 Sendagi, Bunkyo-ku, Tokyo, 113- 8603 Japan; 4 Department of Hygiene and Public Health, Nippon Medical School, 1-1-5 Sendagi, Bunkyo-ku, Tokyo, 113- 8603 Japan

**Keywords:** Heart failure, Pimobendan, GDMT

## Abstract

**Background:**

Pimobendan reportedly improves the subjective symptoms of heart failure. However, evidence of improved prognosis is lacking. This study aimed to determine whether reinforcing guideline-directed medical therapy (GDMT) improved rehospitalization rates for worsening heart failure in patients administered pimobendan.

**Methods:**

A total of 175 patients with heart failure who were urgently admitted to our hospital for worsening heart failure and who received pimobendan between January 2015 and February 2022 were included. Of the 175 patients, 44 were excluded because of in-hospital death at the time of pimobendan induction. The remaining 131 patients were divided into two groups, the reduced ejection fraction (rEF) (*n* = 93) and non-rEF (*n* = 38) groups, and further divided into the GDMT-reinforced and non-reinforced groups.

**Results:**

In patients with rEF, the rate of rehospitalization for heart failure was significantly lower in the GDMT-reinforced group than in the non-reinforced group (log-rank test, *P* = .04). However, the same trend was not observed in the non-rEF group.

**Conclusions:**

Reinforcing GDMT may reduce the heart failure rehospitalization rate in patients with pimobendan administration and rEF. However, multicenter collaborative research is needed.

**Trial registration:**

IRB Approval by the Nippon Medical School Hospital Ethics Committee B-2021-433 (April 10, 2023).

**Supplementary Information:**

The online version contains supplementary material available at 10.1186/s40780-024-00346-w.

## Background

Heart failure is a global epidemic characterized by high prevalence and mortality rates [1, 2]. Rapid advancements have been made in drug therapy for heart failure in recent years, and guideline-directed medical therapy (GDMT)-including β-blockers, mineralocorticoid receptor antagonists (MRA), sodium glucose cotransporter-2 (SGLT2) inhibitors, and angiotensin receptor blockers/angiotensin converting enzyme inhibitors/angiotensin receptor neprilysin inhibitor (ARNI), is strongly recommended. However, few patients receive adequate treatment in accordance with GDMT [3]. The low induction rate of GDMT may be due to various reasons, including patients’ intolerance to the introduction or escalation of drug therapy, renal impairment, hypotension, bradycardia, dehydration, and noncompliance. Pimobendan, a drug known for leading to improvements in subjective symptoms in heart failure patients, lacks evidence supporting its efficacy in improving prognosis, such as reducing rehospitalization rates due to exacerbation of heart failure, Pimobendan functions primarily by inhibiting phosphodiesterase (PDE) III, which produces inotropic effects independent of β-receptor mediation. The Ca-sensitizing effect of pimobendan produces inotropic effects without increasing myocardial oxygen consumption [4, 5]. In practice, there are a certain number of heart failure patients taking pimobendan, and the clinical question remains whether strengthening GDMT improves heart failure rehospitalization rates [6, 7]. In this study, we used single-center registry data to determine whether intensifying GDMT in patients with severe heart failure receiving pimobendan would reduce rehospitalization rates.

## Methods

### Study design and data collection

This single-site cohort study was based on information extracted from the diagnosis procedure combination (DPC) database of the Nippon Medical School Hospital. The following data were extracted: patient age and sex, primary diagnoses and comorbidities, diagnoses and treatments, medications and devices used, discharge status, and post-discharge outcomes. Diagnoses were defined according to the International Classification of Diseases 10th revision (ICD-10). Laboratory data, electrocardiogram, and echocardiographic parameters were collected electronically from the medical records. Data were anonymized and not used to identify individuals. As a result, patient consent was not required. This study was conducted after obtaining permission from the Ethics Committee of the Nippon Medical School Hospital (B-2021-433).

### Patient selection and evaluation

The inclusion period was from January 2015 to February 2022. The inclusion criteria were urgent admission to our hospital for heart failure and treatment with pimobendan. The exclusion criterion was in-hospital death at the time of pimobendan induction. Following the current guidelines, β-blocker or MRA reinforcement refers to patients in whom β-blocker or MRA was increased after pimobendan induction. First, we divided the patients into two groups according to the left ventricular ejection fraction during hospitalization to evaluate the clinical characteristics and readmission rates: the reduced ejection fraction (rEF) and non-rEF (midrange [mr] EF and preserved [p] EF) groups. Second, we further divided the rEF and non-rEF groups into two groups according to the GDMT reinforcement and non-reinforcement (supplemental Figure). Finally, we identified independent factors associated with lower readmission rates. The rEF group was defined as patients with as an EF < 40%, and the non-rEF group was defined as those with an EF ≥ 40% in the echocardiographic evaluation at the time of pimobendane induction.

### Outcomes

The primary endpoint of this study was the rate of rehospitalization due to heart failure within 1 year, defined as readmission with a primary diagnosis of heart failure according to the ICD-10 codes. The secondary endpoint was the Cox univariate model for heart failure rehospitalization within 1 year in the heart failure group.

### Statistical analysis

Categorical variables were expressed as numbers and percentages and compared among the groups using the Chi-squared test. Continuous variables were expressed as means and standard deviations or medians and interquartile ranges. Heart failure readmission rates were compared using the Kaplan–Meier method, and differences were compared using the log-rank test. Cox regression analysis was used to identify independent factors associated with lower readmission rates. Two-sided *P* values of < 0.05 were considered statistically significant. Statistical analyses were performed using SPSS version 28 (IBM Corp., Armonk, NY, USA) and the R software version 4.2.1 (R Foundation for Statistical Computing, Vienna, Austria).

## Results

A total of 175 patients with heart failure who were urgent admitted to our hospital for worsening heart failure and received pimobendan between January 2015 and February 2022 were included in the study. Of the 175 patients, 44 were excluded from this study due to in-hospital deaths at the time of pimobendan induction. The remaining 131 patients were divided into two groups: the rEF group (*n* = 93) and the non-rEF group (*n* = 38), and further divided into the GDMT-reinforced and non-reinforced groups (Table 1, Supplemental Figure). In the rEF group, the GDMT-reinforced group was younger than the non-reinforced group, had a higher MRA induction rate, and had a higher estimated glomerular filtration rate (eGFR), at admission and discharge and Hb at admission. In the non-rEF group, the GDMT-reinforced group had a higher β-blocker and PPI induction rate than the non-reinforced group.

**Table 1:** Background data of patients in the four groups

Variable	HFrEF				non-rEF(HFmrEF + HFpEF)				
	GDMT-reinforced (*n* = 37)	GDMT non-reinforced (*n* = 56)	*p*.value		GDMT-reinforced (*n* = 12)	GDMT non-reinforced (*n* = 26)	*p*.value		
Age (years)	68 (59–78)	74.5 (67.8–81)	0.044		78 (74.3–82.8)	80 (73.3–85)	0.637		
Male, n (%)	29 (78.4)	40 (71.4)	0.481		4 (33.3)	11 (42.3)	0.728		
BMI (kg/m^2^)	22.3 (19.5–25.4)	23.4 (19.2–25.4)	0.900		23.6 (21.5–24.5)	20 (18.2–24.7)	0.177		
PM.ICD, n (%)	15 (40.5)	24 (42.9)	1.000		2 (16.7)	5 (19.2)	1.000		
ACE.ARB, n (%)	32 (86.5)	44 (78.6)	0.417		9 (75.0)	18 (69.2)	1.000		
MRA, n (%)	33 (89.2)	32 (57.1)	0.001		9 (75.0)	13 (50.0)	0.178		
ARNI, n (%)	1 (2.7)	5 (8.9)	0.397		2 (16.7)	0 (0.0)	0.094		
PPI, n (%)	30 (81.1)	41 (73.2)	0.460		6 (50.0)	22 (84.6)	0.045		
β-blocker, n (%)	37 (100.0)	50 (89.3)	0.078		12 (100.0)	16 (61.5)	0.016		
Statin, n (%)	22 (59.5)	33 (58.9)	1.000		7 (58.3)	9 (34.6)	0.289		
Digitalis, n (%)	14 (37.8)	17 (30.4)	0.505		3 (25.0)	4 (15.4)	0.656		
β-blocker or MRA reinforcement, n (%)	25 (67.6)	10 (17.9)	< 0.001		10 (83.3)	4 (15.4)	< 0.001		
Anticoagulant, n (%)	23 (62.2)	34 (60.7)	1.000		8 (66.7)	20 (76.9)	0.694		
Antiplatelet drug, n (%)	22 (59.5)	26 (46.4)	0.290		4 (33.3)	9 (34.6)	1.000		
Diuretic, n (%)	37 (100.0)	53 (94.6)	0.273		12 (100.0)	25 (96.2)	1.000		
NT-proBNP (pg/mL) at discharge	13,147 (2502–23,996)	6702 (3669–14,924)	0.432		5218 (3680–8180)	3847 (1723–6794)	0.243		
Albumin									
At admission (g/dL)	3.5 (3.2–3.9)	3.6 (3.3–3.8)	0.879		3.4 (3.1–3.9)	3.5 (3.2–3.7)	0.875		
At discharge (g/dL)	3.3 (2.9–3.9)	3.6 (3.2–3.7)	0.735		2.9 (2.6–3.4)	3.2 (2.7–3.7)	0.250		
eGFR									
At admission (mL/min/1.73m^2^)	38 (25–55)	29 (19-44.5)	0.021		26.5 (19.3–40.3)	25 (20-37.5)	0.925		
At discharge (mL/min/1.73m^2^)	43 (28–63)	31.5 (22–49)	0.023		34.5 (16.5–51.3)	29 (21-41.8)	0.950		
Hemoglobin									
At admission (g/dL)	12.5 (11.3–14)	11.1 (9.8–12.9)	0.004		11.2 (10-12.5)	10.7 (8.8–11.7)	0.414		
At discharge (g/dL)	10.9 (9.8–11.8)	10.5 (9.4–12.3)	0.296		9.0 (8.4–10.2)	10.1 (9.2–11.4)	0.068		
Systolic blood pressure (mmHg) at discharge	100 (93–106)	103 (95–111)	0.141		108 (101.5-118.7)	102 (98.3–109)	0.176		

Abbreviations: ACE-I, angiotensin converting enzyme inhibitor; ARB, angiotensin receptor blocker; ARNI, angiotensin receptor neprilysin inhibitor; BMI, Body Mass Index; eGFR, estimated glomerular filtration rate; GDMT, Guideline-directed medical therapy; HFmrEF, heart failure with mid-range ejection fraction; HFpEF, heart failure with preserved ejection fraction; HFrEF, heart failure with reduced ejection fraction; ICD, Implantable Cardioverter Defibrillator; MRA, mineralocorticoid receptor antagonist; NT-proBNP, N-terminal pro-brain natriuretic peptide; PM, PaceMaker; PPI, proton pump inhibitor

Continuous data are presented as mean values (standard deviation)

Categorical variables are expressed as numbers and percentages; these were compared among the groups using the chi-square test. Continuous variables are expressed as means and standard deviations or as medians and interquartile ranges (IQRs).

### Post-discharge readmission for heart failure

**Fig. 1:**
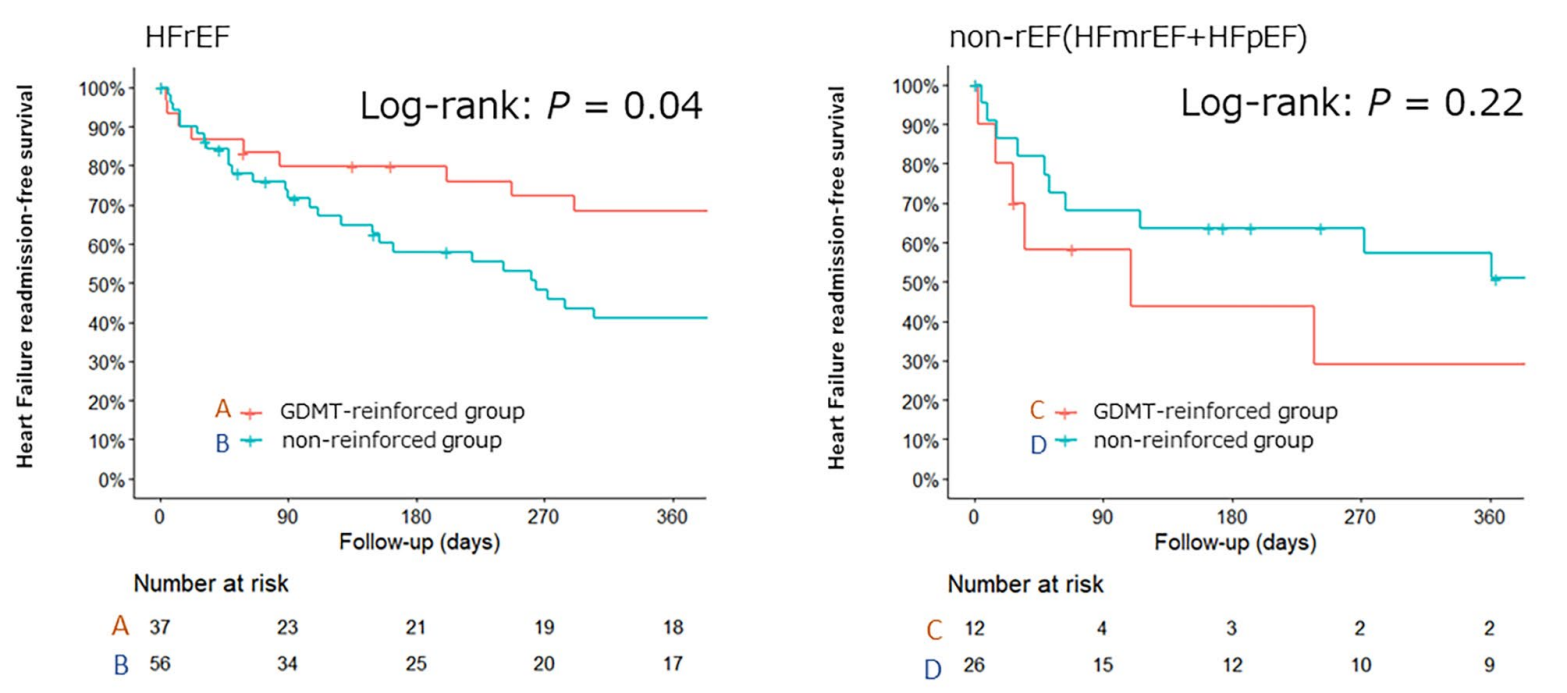
Kaplan Meier curves showing heart failure readmission-free survival in the heart failure with reduced ejection fraction (HFrEF) and non-rEF (heart failure with midrange (mr)EF and preserved (p)EF) groups

The rehospitalization rate for heart failure within 1 year was significantly lower in the GDMT-reinforced rEF group than in the non-reinforced rEF group (log-rank test, *P* = .04) (Fig. 1). Univariate Cox regression analysis identified β-blocker use or MRA reinforcement as factors independently associated with the 1-year readmission rate (Table 2). No significant factors were identified in the multivariate analysis, included of sex, age, eGFR, and GDMT reinforcement (Table 2). β-blocker or MRA reinforcement refers to patients in whom β-blocker or MRA was increased after pimobendan induction. In contrast, there were no significant differences between the GDMT-reinforced and non-reinforced groups in the non-rEF group (log-rank test, *P* = .22) (Fig. 1).

**Table 2:** Cox univariate and multivariate model for heart failure rehospitalization within 1 year in the heart failure with reduced ejection fraction group

Variable	univariate		multivariate	
	Hazard ratio (95% CI)	*p*-value	Hazard ratio (95% CI)	*p*-value
Male	0.95 (0.46–1.97)	0.883	1.15 (0.54–2.47)	0.718
Age	1.02 (0.99–1.04)	0.274	1.01 (0.98–1.04)	0.671
β-blocker or MRA reinforcement	0.47 (0.22–0.99)	0.048	0.54 (0.24–1.21)	0.134
Albumin (g/dL) at discharge	0.84 (0.41–1.71)	0.632		
eGFR (mL/min/1.73m^2^) at discharge	0.98 (0.96-1.00)	0.116	0.99 (0.97–1.01)	0.134
Hemoglobin (g/dL) at discharge	0.92 (0.77–1.10)	0.354		

Abbreviations: eGFR, estimated glomerular filtration rate; MRA, mineralocorticoid receptor antagonist

### Adverse events associated with discontinuation or dose reduction of pimobendan

Among the study patients in the combined rEF and non-rEF groups, the incidence of cardiovascular-related adverse events was 6.9%: six cases of ventricular tachycardia, one case of ventricular fraction-related death, one case of atrial tachycardia, and one case of hypotension. The rate of sudden death from ventricular arrhythmias was one case (0.76%, *n* = 1) in the rEF group.

## Discussion

This study investigated the usefulness of GDMT reinforcement in patients treated with pimobendan, rather than evaluating the efficacy of pimobendan. This study showed that, in patients receiving pimobendan, the rate of heart failure rehospitalization was reduced by reinforcement with GDMT after pimobendan induction in the rEF group. In addition, reinforcement of β-blockers or MRA was identified as a factor influencing the reduction in heart failure rehospitalization in the rEF group, but not multivariate analysis. In contrast, age, albumin at discharge, eGFR, and Hb were not. GDMT did not impact rehospitalization rates for heart failure in the non-rEF group. Both Pimobendan in Congestive Heart Failure (PICO) trial and Effects of Pimobendan on Chronic Heart Failure (EPOCH) study, which were multicenter studies investigating the efficacy of pimobendan, did not include patients using MRA. Additionally, the number of patients using β-blockers was low (22.5% of all patients in the EPOCH study and none of the patients in the PICO trial) [6, 7]. Our study is novel in this respect as we evaluated reinforcement with GDMT in patients receiving pimobendan.

Unlike other inotropic drugs, pimobendan exhibits inotropic effects that are not mediated by β-receptors [8]. As such, this drug is thought to be compatible with β-blockers, and some reports support the safe administration of β-blockers in patients with heart failure [9–11]. Furthermore, the benefit of MRAs in patients with heart failure has been demonstrated in a randomized aldactone evaluation study (RALES) trial, and pimobendan may reduce heart failure rehospitalization rates by enabling MRA induction [12]. The increase in circulating plasma volume after pimobendan induction may increase the tolerability of MRA induction and dose escalation. Pimobendan has also been reported to improve myocardial diastolic dysfunction [13, 14].

Although there have been reports of arrhythmia-related side effects associated with pimobendan, such as in the Prospective Randomized Milrinone Survival Evaluation (PROMISE) study, which found that PDE III inhibitors may induce arrhythmias, there were no such reports from the PICO trial, the EPOCH study, or other reports [4, 15–17]. In the present study, the rate of sudden death due to ventricular arrhythmias was 0.76%, suggesting that pimobendan had a negligible effect on lethal arrhythmias.

This study demonstrates that in patients receiving pimobendan, the heart failure rehospitalization rate was reduced by reinforcement with GDMT after pimobendan induction in the rEF group, but not in the non-rEF group.

### Limitations

This study has several limitations. First, the single-center DPC database design may have introduced selection bias. Second, the study did not include patients who were administered SGLT2 inhibitors and the rate of introduction of ARNI was low, indicating a low rate of introduction of the latest GDMTs as they were not adopted in our hospital during the study period. Third, patients who received pimobendan were more likely to have severe heart failure. It is possible that patients with a poor prognosis were initially included in the study.

## Conclusion

In patients with rEF, the rate of rehospitalization for heart failure was significantly lower in the GDMT-reinforced than nonreinforced group, but not in the non-rEF group. Multicenter collaborative research is therefore needed.

### Electronic supplementary material

Below is the link to the electronic supplementary material.

Supplementary Material 1

## Data Availability

Not applicable.

## Abbreviations

ARNI: angiotensin receptor neprilysin inhibitor
DPC: diagnosis procedure combination
eGFR: estimated glomerular filtration rate
EPOCH: Effects of Pimobendan on Chronic Heart Failure
GDMT: guideline-directed medical therapy
Hb: hemoglobin
ICD-10: International Classification of Diseases 10th revision
MRA: mineralocorticoid receptor antagonists
PDE: phosphodiesterase
PICO: Pimobendan in Congestive Heart Failure
PROMISE: Prospective Randomized Milrinone Survival Evaluation
RALES: randomized aldactone evaluation study
rEF: reduced ejection fraction
SGLT2: sodium glucose cotransporter-2
