# Supplementary figures and images for: Reinforcement of pimobendan with guideline-directed medical therapy may reduce the rehospitalization rates in patients with heart failure: retrospective cohort study
Source: J Pharm Health Care Sci. 2024 May 20;10:24. doi: 10.1186/s40780-024-00346-w (PMC11103862; doi:10.1186/s40780-024-00346-w)

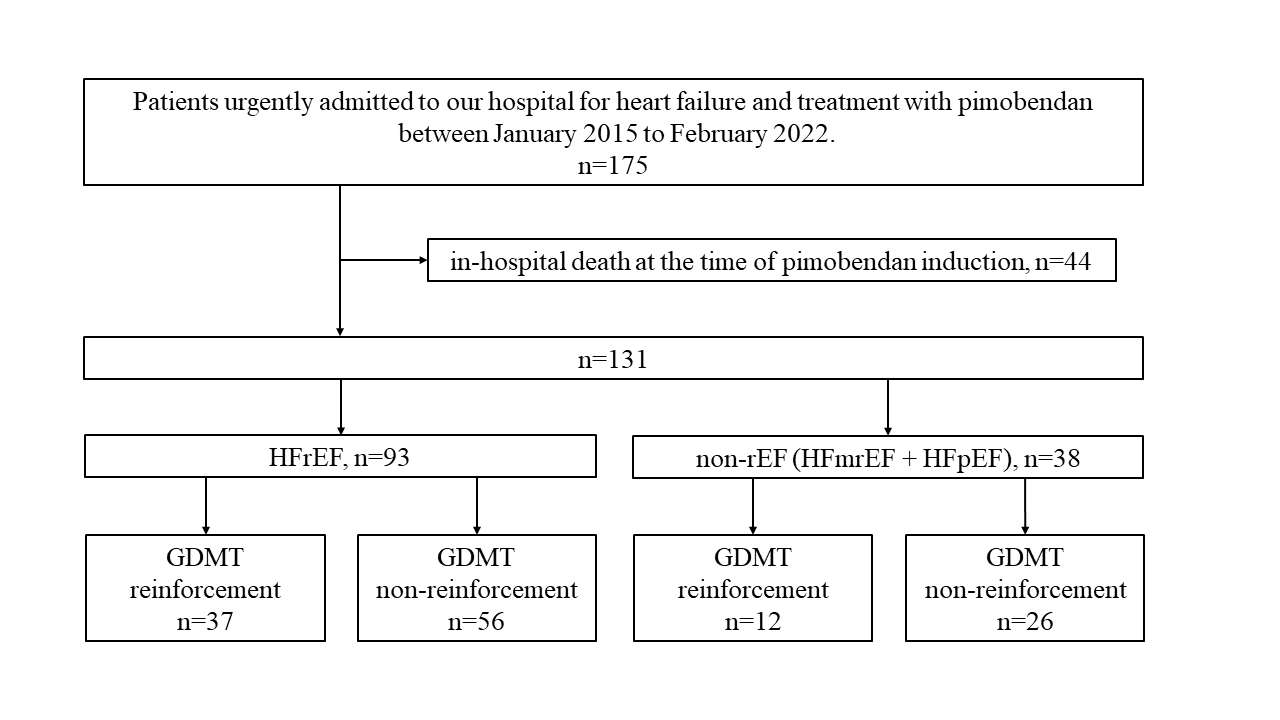

Supplement: Supplementary file 1 — Supplementary Material 1 [file 40780_2024_346_MOESM1_ESM.docx]
